# Mortality rate of mental disorder trends in China from 2002 to 2020

**DOI:** 10.3389/fpsyt.2022.1039918

**Published:** 2022-11-15

**Authors:** Boxuan Li, Guoshuang Zhang, Jing Ma, Mingxiu Kang

**Affiliations:** Tianjin Anding Hospital, Mental Health Center of Tianjin Medical University, Tianjin, China

**Keywords:** mental disorder, mortality rate, risk factor, population distribution, regional distribution

## Abstract

**Background:**

The number of people with mental disorders is increasing in China, but there are few studies on the temporal trends and population distribution of mental disorder mortality.

**Methods:**

The mortality of mental disorders were derived from the China Health Statistics Yearbook published by the National Health and Family Planning Commission. Temporal trends in mortality were examined with a joinpoint regression using annual percent change (APC) and average annual percent change (AAPC). A Poisson regression model was utilized to test the population-level risk factors associated with the death of people with mental disorders.

**Results:**

The mortality of mental disorders in rural Chinese residents showed a decreasing trend from 2002 to 2020 [AAPC –2.06%, 95% confidence interval (CI) –3.16 to –0.91%]. The mortality of mental disorders in urban Chinese residents declined between 2005 and 2011 (APC –13.01%, 95% CI –21.08 to –4.13%). The mortality rate of mental disorders has decreased for urban males with an APC of –2.71% (95% CI –4.52 to –0.71) from 2002 to 2020. Urban women showed an increase in mental disorder mortality from 2002 to 2005 and from 2012 to 2020 with APCs of 19.65% (95% CI 0.64–42.32%) and 6.16% (95% CI 2.22–10.33%), respectively. Age was a significant risk factor for mental disorder mortality (odds ratio 1.28, 95% CI 1.23–1.32).

**Conclusion:**

The dissemination of medical and health information, investment in medical and health resources, and the modification and optimization of regulations have led to a decrease in mental disorder mortality in China. It is vital to devote greater attention to elderly individuals suffering from mental disorders.

## Introduction

In the 10th Edition of the International Statistical Classification of Diseases and Related Health Problems, mental disorders are defined as “a clinically recognizable set of symptoms or behaviors associated in most cases with distress and with interference with personal functions” (1), which are among the leading causes of worldwide health-related burdens (2). According to the Global Burden of Disorders, Injuries, and Risk Factors, depression and anxiety disorders are the two that most reduce quality of life, ranking among the top 25 leading causes of burden globally in 2019 (2). Due to the traits of insidious onset, prolonged symptoms, treatment delays, and recurrent course, the prognosis of mental disorders are often unfavorable (3–6). Patient daily life may be affected and even distressed (7). Many studies have shown that some, if not most, patients seek informal medical care after onset of their illness, leading to misdiagnosis, delayed treatment, accelerated disease progression, and death (4, 8, 9).

With the rapid development of China, social and work pressures are intensifying, contributing to a rise in mental health issues. As with the general situation in the world, the number of people with mental disorders in China is gradually increasing as a result of rapid population growth and the serious problem of population aging (10). Furthermore, the prevalence of the risk factors of mental disorders in Chinese population has increased, such as migration, smoking, alcohol use, and physical inactivity (11, 12), causing a larger amount of patients with mental disorders. Moreover, with its large population, the treatment of mental disorders is highly relevant in China. The China Mental Health Survey reported a 12-month prevalence of mental disorders (except dementia) was 4.1% [95% confidence interval (CI) 3.4–4.7] and life-time prevalence was 7.4% (95% CI 6.3–8.4) in adults (13–15). Although some researchers have analyzed the mortality of mental disorders in China, there have been few discerptions of its temporal trends (16, 17).

The aim of the present study was to analyze and summarize the mortality rate of mental disorders and associated risk factors among Chinese residents from 2002 to 2020 in order to explore the changing trends and influencing factors of mental disorder mortality and provide a scientific foundation for future work on disease prevention.

## Materials and methods

### Data sources

This study used data from the China Health Statistical Yearbook, 2002–2020 (renamed China’s Health and Family Planning Statistical Yearbook after 2013) to analyze the mortality of mental disorders in China. Data were collected from the Statistical Information Center of the National Health and Wellness Commission of the People’s Republic of China, whose results are based on an approximate 10% of the population in 31 provinces across China (18). Data were obtained from the cause of death reporting system for patients who died at all levels of medical institutions in China. The cause of death was determined by qualified clinicians, and diagnoses included in this study were from the mental and behavioral disorders section of the ICD-10 (F00-F99). Based on urban and rural areas (two strata), sex (two strata), and 5-year interval age groups (18 strata), the yearbook provides statistics on mortality attributable to mental disorders for each of the 72 strata of the population. Additionally, the yearbook contains death rates for mental diseases for 36 strata of the population, divided by sex and age. China implemented ICD-10 for the first time in 2002. The present study utilized the 2002–2020 China Health Statistical Yearbook results in order to establish the comparability of longitudinal data. Since the data were derived from the Health and Family Planning Statistical Yearbook of China, no ethical approval was required.

### Statistical analysis

The crude mortality rate of mental disorders obtained directly from the China Health Statistical Yearbook is called the mortality rate of mental disorders, and the direct standardization method was used to standardize the mortality rate of mental disorders based on age. The age groups in the present study population spanned 5 years, and there were 16 age groups in total. A total of 64 strata of the population was obtained after considering the factors of urban/rural habitation and sex.

The joinpoint method was used to calculate the annual percent change (APC) and average annual percent change (AAPC) of mortality for mental disorders (19). Joinpoint Regression Software was used to fit the Joinpoint model (Version 4.9.1.0) (20). A joinpoint regression model is designed to identify a combination of trends that provides a statistically significantly better fit for a data series than a single-trend line fitted with a Poisson regression model or a time series model. Using this procedure, it is possible to determine the number of joinpoints that are necessary to assess significant changes in incidence trends over time. This technique is used to identify the calendar year (joinpoint) in which statistically significant abrupt changes in temporal trends occurred (21). The number and locations of joinpoints and the corresponding *p*-values were determined by Monte Carlo permutation tests, and the model fit was tested using a Bayesian information criterion. According to the joinpoint model, *P*-values < 0.05 indicated statistical significance of the difference between the joinpoints. In the absence of a joinpoint (joinpoint = 0), APC = AAPC, indicating an overall monotonic trend in mortality. APC > 0 indicated that the mortality rate was increasing over time; an APC < 0 indicated a decrease over time.

The Poisson regression model was to test the population-level risk factors associated with the death of people with mental disorders from 2002 to 2020. The dependent variable was the observed mortality rate in the observation unit, and the risk factor indicators were region (urban/rural), sex (male/female), age, and year of observation. The regression analysis examined only the risk factors for death after age 20, as there was a low mortality rate for mental disorders in the younger age groups.

## Results

### Age-standardized mortality rates of mental disorders

The mortality rate of mental disorders decreased for both men and women between 2002 and 2020, and it was higher in rural than urban areas and for women than for men (Table 1).

**TABLE 1:** Age-standardized mortality rate of mental disorder for Chinese residents from 2002 to 2020/10^5^.

Year	Urban		Rural	
	Male	Female	Male	Female
2002	3.06	2.93	3.98	4.60
2003	3.10	3.08	3.65	4.31
2004	3.12	2.87	2.91	3.29
2005	5.44	5.88	2.39	3.15
2006	2.80	3.43	3.88	4.34
2007	3.66	4.06	3.44	4.06
2008	2.30	2.56	3.88	4.73
2009	2.56	2.58	2.73	3.47
2010	2.52	2.54	3.58	4.25
2011	1.81	1.87	3.21	3.79
2012	1.52	1.37	2.95	2.97
2013	2.26	2.09	2.64	2.82
2014	2.15	1.95	2.54	2.59
2015	2.47	2.64	2.83	3.40
2016	2.30	2.51	2.68	2.91
2017	2.29	2.48	2.6	2.83
2018	2.32	2.53	2.54	2.76
2019	2.33	2.64	2.64	3.16
2020	2.24	2.41	2.73	3.05

### Temporal trends of age-standardized mortality in mental disorders

Based on the mortality rate of mental disorders among Chinese urban residents from 2002 to 2020, the APC of 0.31% (95% CI –4.31–5.07%) was not statistically significant (*P* = 0.906). Nevertheless, joinpoint regression revealed a statistically significant reduction in urban resident mental disorder mortality of 13.01% (95% CI –21.08 to –4.13%) between 2005 and 2011, followed by an increasing but not statistically significant trend from 2011 to 2020 (APC = 3.57%, 95% CI –0.52–7.78%, *P* = 0.079). The joinpoint regression analysis of urban resident revealed an AAPC of –2.71% in men (95% CI –4.52% to –0.71%, *P* = 0.010) and an AAPC of 0.41% for women (95% CI –3.10–4.07%, *P* = 0.811). In addition, urban women showed significantly increasing trends in mental disorder mortality from 2002 to 2005 (APC 19.65%, 95% CI 0.64–42.32%, *P* = 0.044) and from 2012 to 2020 (APC 6.16%, 95% CI 2.22–10.33%, *P* = 0.005). A decline was observed between 2005 and 2012 (APC –12.53% 95% CI –17.52 to –7.21%, *P* < 0.001).

**FIGURE 1:**
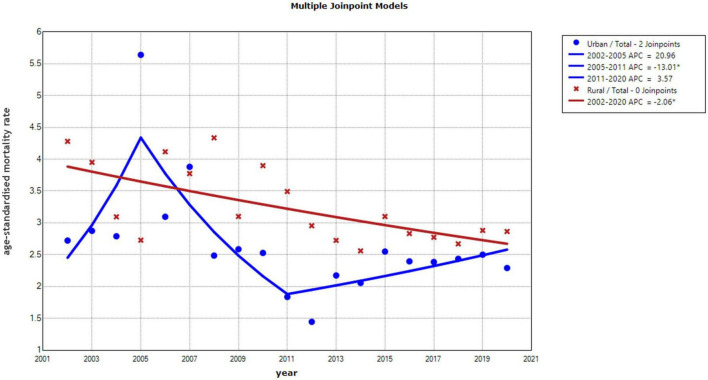
Annual percentage change (APC) in the trend of age-standardized mortality rates for Chinese residents by region from 2002 to 2020.

**FIGURE 2:**
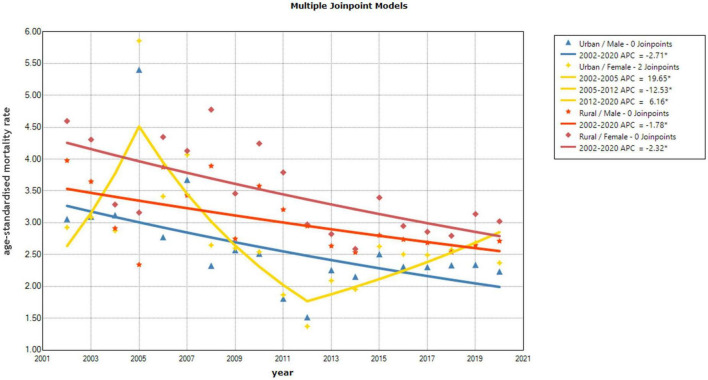
Annual percentage change (APC) in the trend of age-standardized mortality rates for Chinese residents by gender from 2002 to 2020.

Mental disorder mortality in rural residents declined by 2.06% annually (95% CI –3.16 to –0.91%, *P* = 0.002). For men, the average annual decline in mortality rates from 2002 to 2020 was 1.78% (95% CI –2.97 to –0.62%, *P* = 0.006), and for women it was 2.32% (95% CI –3.51 to –1.13%, *P* = 0.001) (Figures 1, 2).

### Trends in mental disorders by age group

We used joinpoint regression to determine the mortality rates of mental diseases at different ages. The mortality trends for each age group in the four populations were not constant, but there were some significant turning points. Except for the 40–44, 50–54, 60–64, 65–69, and 80–84 age groups, the mortality rate of mental disorders among urban males decreased year by year in all other age groups. Among them, the 30–34-year-old and 35–39-year-old age groups experienced the greatest decrease in mental disorder mortality, with AAPC of –9.92 and –9.19%, respectively. Among urban women, the mortality rate of mental disorders in the age groups 64–69 and over 70 did not demonstrate a significant decrease trend, but the mortality rates of mental disorders in the other age groups showed a decreasing tendency over time. Similar to urban males in the 30–34 age group, urban women in the 30–34 age group revealed the most significant decline in death from mental disorders, with an AAPC of 8.54%. Males in rural areas exhibited more heterogeneity in mental disorder mortality across age groups, but also the highest decrease in the 30–34 and 35–39 age groups, with AAPC values of –7.60 and –7.09%, respectively. Among rural women, the 35–39 age group had the greatest AAPC at –11.11% (Table 2).

**TABLE 2:** Annual changes in mortality rates of mental disorder for Chinese residents from 2002 to 2020.

Age group (year)	Urban				Rural			
	Male		Female		Male		Female	
	APC (%)	AAPC (%)	APC (%)	AAPC (%)	APC (%)	AAPC (%)	APC (%)	AAPC (%)
20–	–5.26*	–5.26*	–10.18*	–10.18*	–3.94*	–3.94*	–8.30*	–8.30*
25–	–6.25*	–6.25*	2002–2008: –18.51*	–11.10*	–2.44	–2.44	2.80	2.80
			2008–2017: 3.88					
			2017–2020: –33.73					
30–	–9.92*	–9.92*	–8.54*	–8.54*	2002–2007: –23.04*	–7.60*	–4.51*	–4.51*
					2007–2020: –0.85			
35–	–9.19*	–9.19*	–7.58*	–7.58*	–7.09*	–7.09*	–11.11*	–11.11*
40–	2002–2004: 41.98	–3.50	–6.35*	–6.35*	2002–2012: 2.99	–3.40	0.34	0.34
	2004–2020: –8.02*				2012–2020: –10.91*			
45–	–5.25*	–5.25*	–4.08*	–4.08*	2002–2004: –32.58	–4.10	–4.12*	–4.12*
					2004–2020: 0.25			
50–	2002–2007: 1.32	–2.50	–6.54*	–6.54*	2002–2004: 30.57	–1.60	–3.02*	–3.02*
	2007–2012: –23.97*				2004–2012: –13.60*			
	2012–2015: 40.68				2012–2017: 13.73			
	2015–2020: –11.19				2017–2020: –11.92			
55–	–3.67*	–3.67*	–3.66*	–3.66*	–3.97*	–3.97*	–5.36*	–5.36*
60–	2.07	2.07	–2.15*	–2.15*	1.17	1.17	2002–2005: –23.01	–3.9
							2005–2020: 0.49	
65–	–1.15	–1.15	2002–2011: –9.93*	–2.70	–0.70	–0.70	2002–2004: –34.69	–4.9
			2011–2020: 5.08				2004–2020: –0.32	
70–	–4.64*	–4.64*	2002–2005: 18.38	–2.30	–2.16	–2.16	–3.15*	–3.15*
			2005–2011: –17.56*					
			2011–2020: 2.62					
75–	–3.77*	–3.77*	2002–2005: 18.23	–2.70	–2.27	–2.27	–5.31*	–5.31*
			2005–2012: –14.13*					
			2012–2020: 0.90					
80–	–0.81	–0.81	2002–2005: 33.25*	1.20	–0.97	–0.97	–2.53*	–2.53*
			2005–2012: –14.62*					
			2012–2020: 6.02*					
85–	4.34*	4.34*	2002–2005: 22.93	4.40	2.55	2.55	2002–2004: –24.58	–0.40
			2005–2012: –10.28*				2004–2007: 30.38	
			2012–2020: 12.08*				2007–2020: –2.27	
Total	–2.71*	–2.71*	2002–2005: 19.65*	0.41	–1.78*	–1.78*	–2.32*	–2.32*
			2005–2012: –12.53*					
			2012–2020: 6.16					

APC, annual percent change; AAPC, average annual percent change. *P < 0.05.

### Risk factors of mental disorder mortality for Chinese residents

Table 3 shows the results of the Poisson regression model used to estimate the mental disorder mortality rate for Chinese residents. Its good fit is characterized by deviations of 68.15, scaling deviations of 68.15, and Pearson coefficients of 82.37, with 1,439 degrees of freedom in the model (*P* > 0.05). In terms of mortality, both age and year were independent risk factors. The risk of death from mental disorders increased by 28% (95% CI 1.23–1.32, *P* < 0.0001) per 5 years of age in the same sex and region. Each year, the risk of death decreased by 3% (95% CI 0.95–0.99, *P* = 0.0369) (Table 3).

**TABLE 3:** The risk factors in mental disorders deaths for Chinese residents from 2002 to 2020.

Factor	Parameter estimate	OR	OR 95% CI	*P*-value
Region	–0.1909	0.83	0.64–1.07	0.152
Gender	–0.0973	0.91	0.70–1.18	0.4639
Age	0.2446	1.28	1.23–1.32	<0.0001
Year	–0.0254	0.97	0.95–0.99	0.0369

## Discussion

This study analyzed temporal trends in mental disorder mortality in China from 2002 to 2020 based on official data released by the China Health and Welfare Commission. From 2002 to 2020, mental disorder mortality decreased overall among rural Chinese residents, and mental disorder mortality among urban Chinese residents decreased overall from 2005 to 2011, with no statistically significant changes noted in other years. Age was associated with an increase in mortality from mental disorders.

Currently, China has two sources of cause of death data, the first from disease surveillance points (22), and the second from the National Health Commission (23), which was used in the present study. The cause-of-death surveillance information from the disease surveillance points system is unsuitable for time-trend analysis due to a lack of data on urban-rural, sex-, and age-specific mortality rates, as well as possible inconsistencies between the years (24). Since 2002, China has used the ICD-10 classification system for mental disorders, allowing multi-year trend investigations.

In the present study, the mortality rate of patients with mental disorders decreased or remained stable. Despite the increase in the number and prevalence of mental disorders in China (10), the mortality rate has not increased, which may be due to China’s mental health programs, such as the “Central Government Support for the Local Management and Treatment of Severe Mental Illnesses Project” (686 project) (25). The 686 Project was launched in 2004 to provide comprehensive mental health services to patients with severe mental disorders (26). The goal of this project was to improve medication adherence by routinely and conveniently providing free medications to patients suffering from severe mental disorders (27, 28). It was included in the daily routine of the project staff to ask members if they had taken their medications. Importantly, the program notes that patients should be referred to as members to reduce stigma and create an environment that facilitates recovery. They should also be encouraged to discuss their results and their practice of taking medications with each other. Furthermore, lectures on medication adherence were conducted periodically in order to improve the recovery process. Based on the findings of Dou et al., patients in the 686 project group adhered to their medications at a much higher rate than those in the control group, suggesting that implementing the 686 project led to a significant increase in medication adherence (29). Positive changes in medication adherence can reduce mortality rate of mental disorders (30).

By using joinpoint regression analysis, it was determined that, from 2002 to 2020, there were three phases of change in the mental disorder mortality rate of urban residents, with a significant decrease between 2005 and 2011 and no statistically significant changes during the remaining years. It was observed that urban men with mental disorders had a declining mortality rate, whereas urban women showed a substantial increase between 2002 and 2005, followed by a substantial decline between 2006 and 2012. In 2005, the Chinese Law on the Protection of Women’s Rights and Interests was revised, which is the first revision of this policy since its promulgation (31). This law provides more comprehensive protection for women’s rights in health, medicine, employment, and other aspects, as well as in the field of mental health (32). In comparison with rural women, urban women may be subject to greater employment pressures (33). Promulgation and implementation of the revised law might significantly improve the living and working conditions of urban women with mental disorders, increase the availability of medical resources, and reduce the mortality rate among those with mental disorders. It is consistent with the observed changes in mortality rates for mental disorders among urban women. Additionally, the economic development of China (34), increased investment in health care (35), and the improvement of nutritional status (36) may have contributed to a reduction in mental disorder mortality.

Liang et al. examined the relationship between mental disorder mortality and suicide mortality in the Chinese population from 2000 to 2014, using the same data sources as the present study (37). The research discovered a declining trend in mental illness mortality from 2000 to 2014, with a higher decline for women than for men and disparities between urban and rural locations, comparable to our findings. However, their research was not standardized using demographic data; therefore, it was not feasible to determine if the individual patterns were statistically significant.

There have also been other factors contributing to the change in mortality from mental disorders in China. It has been suggested that environmental pollutants, such as PM_2_._5_, may contribute to an increase in mortality (38). The Chinese government has implemented policies to control pollution, such as household fuel policies in northern China, resulting in a decrease in PM_2_._5_ concentrations, which may contribute to a decrease in mental disorder mortality (39, 40). Furthermore, since the passage of the Mental Health Law in China, the living conditions of patients with mental disorders have significantly improved, and the number of homeless and unemployed patients has decreased, resulting in a decrease in overall mortality rates (12).

### Strengths

First, to the best of our knowledge, this is the first study to use joinpoint regression to assess patterns in mortality rates of mental diseases. As compared to the methods used in prior research, this technique addresses the analysis of longitudinal mortality change narratives using segmented regression, with data-driven model selection and more precise computations. Second, this study utilized data collected after China adopted the ICD-10 standard, so longer-term trends could be observed. Third, this study standardized the mental disorder mortality rate in China to be more representative of the national situation. This provides important basic information on resource allocation for mental disorder health services.

### Limitations

This study also had some unavoidable limitations. First, the data in this study were taken from the Statistical Yearbook. Data from the yearbook are derived from a sample survey of 8% of the country’s population, and the sample selection may be affected by the quality of the reporting mechanism. As health care advances, however, this issue may have less of an effect on the reality of mental illness-related mortality. Further, due to limited health resources in rural areas, there may be instances of underreporting or misreporting of deaths due to unclear classification of causes of death. In light of the limitations of data availability, this paper analyzes only the relationship between age, gender, region, and the mortality of mental disorders. It is still necessary to explore other possible influencing factors in the future.

## Conclusion

As a result of popularizing medical and health knowledge, investing in medical and health resources for mental disorders, and adjusting and optimizing policies in China, the mortality rate of mental disorders has declined. However, the prevalence of data collection needs to be improved, particularly in rural areas. In addition, age is a risk factor for mental disorders, which suggests that we should pay more attention to the elderly with mental disorders in the future in order to prevent death more effectively and to reduce the disease burden and excess mortality associated with mental disorders.

## Data availability statement

The raw data supporting the conclusions of this article will be made available by the authors, without undue reservation.

## Author contributions

MK made substantial contributions to the design of the study. BL performed the study and wrote the article. GZ collected the data. JM analyzed and interpreted the data. All authors contributed to the article and approved the submitted version.

## References

[1] International Advisory Group for the Revision of Icd-10 Mental and Behavioural Disorders. A conceptual framework for the revision of the ICD-10 classification of mental and behavioural disorders. *World Psychiatry.* (2011) 10:86–92. 21633677 10.1002/j.2051-5545.2011.tb00022.x PMC3104876

[2] Covid-19 Mental Disorders Collaborators. Global prevalence and burden of depressive and anxiety disorders in 204 countries and territories in 2020 due to the COVID-19 pandemic. *Lancet.* (2021) 398:1700–12. 10.1016/S0140-6736(21)02143-7 34634250 PMC8500697

[3] Chen H Wang T Wang D Gao X. Time delay in seeking treatment for first-episode schizophrenia: a retrospective study. *Early Interv Psychiatry.* (2020) 14:553–8. 10.1111/eip.12879 31591818

[4] Bechard-Evans L Schmitz N Abadi S Joober R King S Malla A. Determinants of help-seeking and system related components of delay in the treatment of first-episode psychosis. *Schizophr Res.* (2007) 96:206–14. 10.1016/j.schres.2007.07.017 17719746

[5] Schramm E Klein DN Elsaesser M Furukawa TA Domschke K. Review of dysthymia and persistent depressive disorder: history, correlates, and clinical implications. *Lancet Psychiatry.* (2020) 7:801–12. 10.1016/S2215-0366(20)30099-7 32828168

[6] Weisberg RB. Overview of generalized anxiety disorder: epidemiology, presentation, and course. *J Clin Psychiatry.* (2009) 70(Suppl 2):4–9. 19371500

[7] Liu Z Li P Yin H Li M Yan J Ma C Future trends in disability and its determinants among chinese community patients with anxiety disorders: evidence from a 5-year follow-up study. *Front Psychiatry.* (2021) 12:777236. 10.3389/fpsyt.2021.777236 34955923 PMC8695844

[8] Cui X Li M Li P Li J Hou X Yan G Help-Seeking behaviors and related factors in chinese patients with major depressive disorder: A community-based cross-sectional study. *Front Psychiatry.* (2022) 13:934428. 10.3389/fpsyt.2022.934428 35873223 PMC9298966

[9] Girma E Tesfaye M. Patterns of treatment seeking behavior for mental illnesses in Southwest Ethiopia: a hospital based study. *BMC Psychiatry.* (2011) 11:138. 10.1186/1471-244X-11-138 21859455 PMC3170592

[10] Ferrari A Santomauro D Herrera A Shadid J Ashbaugh C Erskine H Global, regional, and national burden of 12 mental disorders in 204 countries and territories, 1990-2019: a systematic analysis for the Global Burden of Disease Study 2019. *Lancet Psychiatry.* (2022) 9:137–50. 10.1016/S2215-0366(21)00395-3 35026139 PMC8776563

[11] Spaho E Alikaj V Dashi E Skendi V. Migration: A risk factor for psychosis? *Eur Psychiatry.* (2021) 64:S734–734. 10.1192/j.eurpsy.2021.1944

[12] Yang G Wang Y Zeng Y Gao GF Liang X Zhou M Rapid health transition in China, 1990–2010: findings from the Global Burden of Disease Study 2010. *Lancet.* (2013) 381:1987–2015. 10.1016/S0140-6736(13)61097-1 23746901 PMC7159289

[13] Huang Y Liu Z Wang H Guan X Chen H Ma C The China Mental Health Survey (CMHS): I. background, aims and measures. *Soc Psychiatry Psychiatr Epidemiol.* (2016) 51:1559–69. 10.1007/s00127-016-1270-z 27796403

[14] Liu Z Huang Y Lv P Zhang T Wang H Li Q The china mental health survey: II. design and field procedures. *Soc Psychiatry Psychiatr Epidemiol.* (2016) 51:1547–57. 10.1007/s00127-016-1269-5 27803977

[15] Huang Y Wang Y Wang H Liu Z Yu X Yan J Prevalence of mental disorders in China: a cross-sectional epidemiological study. *Lancet Psychiatry.* (2019) 6:211–24. 10.1016/S2215-0366(18)30511-X 30792114

[16] Zhou M Wang H Zeng X Yin P Zhu J Chen W Mortality, morbidity, and risk factors in China and its provinces, 1990–2017: a systematic analysis for the Global Burden of Disease Study 2017. *Lancet.* (2019) 394:1145–58. 10.1016/S0140-6736(19)30427-1 31248666 PMC6891889

[17] Zhou M Wang H Zhu J Chen W Wang L Liu S Cause-specific mortality for 240 causes in China during 1990-2013: a systematic subnational analysis for the Global Burden of Disease Study 2013. *Lancet.* (2016) 387:251–72. 10.1016/S0140-6736(15)00551-6 26510778

[18] Yang G Hu J Rao KQ Ma J Rao C Lopez AD. Mortality registration and surveillance in China: History, current situation and challenges. *Popul Health Metr.* (2005) 3:3. 10.1186/1478-7954-3-3 15769298 PMC555951

[19] Kim HJ Fay MP Feuer EJ Midthune DN. Permutation tests for joinpoint regression with applications to cancer rates. *Stat Med.* (2000) 19:335–51. 10649300 10.1002/(sici)1097-0258(20000215)19:3<335::aid-sim336>3.0.co;2-z

[20] National Cancer Institute. *Joinpoint Regression Program, Version 4.9.1.0 – April 2022. Surveillance Research Program, Statistical Methodology and Applications Branch*. Bethesda, MD: National Cancer Institute (2022).

[21] Akhtar S Al-Abkal J Alroughani R. Joinpoint regression analysis of trends in multiple sclerosis incidence in kuwait: 1980-2019. *Neuroepidemiology.* (2020) 54:472–81. 10.1159/000511205 33176327

[22] Cheng J Wang W Xu J Yin L Liu Y Wu J. Trends in stroke mortality rate — china, 2004–2019. *China CDC Wkly.* (2022) 4:513–7. 10.46234/ccdcw2022.113 35812696 PMC9257691

[23] Wang Y Li L. Assessment of completeness of Chinese vital registration data. *Chin J Health Stat.* (2007) 04:367–71.

[24] Zhao ZP Wang LM Li YC Jiang Y Zhang M Huang ZJ Provincial representativeness assessment of China Non-communicable and Chronic Disease Risk Factor Surveillance System in 2013. *Zhonghua Yu Fang Yi Xue Za Zhi.* (2018) 52:165–9. 10.3760/cma.j.issn.0253-9624.2018.02.009 29429271

[25] Ma H. Integration of hospital and community services—the ‘686 Project’—is a crucial component in the reform of China’s mental health services. *Shanghai Arch Psychiatry.* (2012) 24:172–4. 10.3969/j.issn.1002-0829.2012.03.007 25324622 PMC4198849

[26] Deng M Zhai S Ouyang X Liu Z Ross B. Factors influencing medication adherence among patients with severe mental disorders from the perspective of mental health professionals. *BMC Psychiatry.* (2022) 22:22. 10.1186/s12888-021-03681-6 34996394 PMC8740063

[27] Liu J Ma H He Y-L Xie B Xu Y-F Tang H-Y Mental health system in China: history, recent service reform and future challenges. *World Psychiatry.* (2011) 10:210–6. 10.1002/j.2051-5545.2011.tb00059.x 21991281 PMC3188776

[28] Xiang Y-T Ng CH Yu X Wang G. Rethinking progress and challenges of mental health care in China. *World Psychiatry.* (2018) 17:231–2. 10.1002/wps.20500 29856546 PMC5980243

[29] Dou L Hu L Zhang N Cutler H Wang Y Li S. Factors associated with medication adherence among patients with severe mental disorders in china: a propensity score matching study. *Patient Prefer Adher.* (2020) 14:1329–39. 10.2147/PPA.S255934 32801663 PMC7402865

[30] Li Y Yan LL Ronsmans C Wen H Xu J Wang D Excess mortality among patients with severe mental disorders and effects of community-based mental healthcare: a community-based prospective study in Sichuan. China. *BJPsych Open.* (2021) 7:e84. 10.1192/bjo.2021.46 33883057 PMC8086393

[31] China Daily. *Law to comprehensively protect women’s rights.* (2005). Available online at: http://en.npc.gov.cn.cdurl.cn/2021-12/23/c_694157.htm (accessed September 2, 2022).

[32] Chen H Chen D. The changing mechanism of the sex difference in life expectancy in china. *Popul Res.* (2022) 46:117–28.

[33] Gignac MAM Ibrahim S Smith PM Kristman V Beaton DE Mustard CA. The Role of Sex. Gender, Health Factors, and Job Context in Workplace Accommodation Use Among Men and Women with Arthritis. *Ann Work Expo Health.* (2018) 62:490–504. 10.1093/annweh/wxx115 29420700 PMC5905635

[34] Byrne P James A. Placing poverty-inequality at the centre of psychiatry. *BJPsych Bull.* (2020) 44:187–90. 10.1192/bjb.2020.85 32981561 PMC7525584

[35] Sørensen CW Bæk O Kallestrup P Carlsson J. Integrating mental health in primary healthcare in low-income countries: changing the future for people with mental disorders. *Nordic J Psychiatry.* (2017) 71:151–7. 10.1080/08039488.2016.1245784 27774828

[36] Melamed O Voineskos A Vojtila L Ashfaq I Veldhuizen S Dragonetti R Technology-enabled collaborative care for youth with early psychosis: Results of a feasibility study to improve physical health behaviours. *Early Interv Psychiatry.* (2022) 16:1143–51. 10.1111/eip.13266 35103380

[37] Liang Y Yang M Zhao G Mao Y Zhang L Hu Z. Relationship between mortality in people with mental disorders and suicide mortality in China during 2000 to 2014: An observational study. *Medicine.* (2018) 97:e13359. 10.1097/MD.0000000000013359 30544403 PMC6310536

[38] Attademo L Bernardini F Garinella R Compton MT. Environmental pollution and risk of psychotic disorders: A review of the science to date. *Schizophr Res.* (2017) 181:55–9. 10.1016/j.schres.2016.10.003 27720315

[39] Meng W Zhong Q Chen Y Shen H Yun X Smith KR Energy and air pollution benefits of household fuel policies in northern China. *Proc Natl Acad Sci USA.* (2019) 116:16773–80. 10.1073/pnas.1904182116 31383761 PMC6708357

[40] Yu G Wang F Hu J Liao Y Liu X. Value assessment of health losses caused by PM2.5 in Changsha City, China. *Int J Environ Res Public Health.* (2019) 16:2063. 10.3390/ijerph16112063 31212685 PMC6604026

